# Intracranial Densitometry-Augmented Machine Learning Enhances the Prognostic Value of Brain CT in Pediatric Patients With Traumatic Brain Injury: A Retrospective Pilot Study

**DOI:** 10.3389/fped.2021.750272

**Published:** 2021-11-02

**Authors:** Young-Tak Kim, Hakseung Kim, Choel-Hui Lee, Byung C. Yoon, Jung Bin Kim, Young Hun Choi, Won-Sang Cho, Byung-Mo Oh, Dong-Joo Kim

**Affiliations:** ^1^Department of Brain and Cognitive Engineering, Korea University, Seoul, South Korea; ^2^Department of Radiology, Massachusetts General Hospital, Boston, MA, United States; ^3^Department of Neurology, Korea University Anam Hospital, Korea University College of Medicine, Seoul, South Korea; ^4^Department of Radiology, Seoul National University Hospital, Seoul National University College of Medicine, Seoul, South Korea; ^5^Department of Neurosurgery, Seoul National University Hospital, Seoul National University College of Medicine, Seoul, South Korea; ^6^Department of Rehabilitation Medicine, Seoul National University Hospital, Seoul National University College of Medicine, Seoul, South Korea; ^7^National Traffic Injury Rehabilitation Hospital, Yangpyeong, South Korea; ^8^Department of Artificial Intelligence, Korea University, Seoul, South Korea

**Keywords:** pediatric traumatic brain injury, computed tomography, densitometric analysis, prognostic modeling, machine learning

## Abstract

**Background:** The inter- and intrarater variability of conventional computed tomography (CT) classification systems for evaluating the extent of ischemic-edematous insult following traumatic brain injury (TBI) may hinder the robustness of TBI prognostic models.

**Objective:** This study aimed to employ fully automated quantitative densitometric CT parameters and a cutting-edge machine learning algorithm to construct a robust prognostic model for pediatric TBI.

**Methods:** Fifty-eight pediatric patients with TBI who underwent brain CT were retrospectively analyzed. Intracranial densitometric information was derived from the supratentorial region as a distribution representing the proportion of Hounsfield units. Furthermore, a machine learning-based prognostic model based on gradient boosting (i.e., CatBoost) was constructed with leave-one-out cross-validation. At discharge, the outcome was assessed dichotomously with the Glasgow Outcome Scale (favorability: 1–3 vs. 4–5). In-hospital mortality, length of stay (>1 week), and need for surgery were further evaluated as alternative TBI outcome measures.

**Results:** Densitometric parameters indicating reduced brain density due to subtle global ischemic changes were significantly different among the TBI outcome groups, except for need for surgery. The skewed intracranial densitometry of the unfavorable outcome became more distinguishable in the follow-up CT within 48 h. The prognostic model augmented by intracranial densitometric information achieved adequate AUCs for various outcome measures [favorability = 0.83 (95% CI: 0.72–0.94), in-hospital mortality = 0.91 (95% CI: 0.82–1.00), length of stay = 0.83 (95% CI: 0.72–0.94), and need for surgery = 0.71 (95% CI: 0.56–0.86)], and this model showed enhanced performance compared to the conventional CRASH-CT model.

**Conclusion:** Densitometric parameters indicative of global ischemic changes during the acute phase of TBI are predictive of a worse outcome in pediatric patients. The robustness and predictive capacity of conventional TBI prognostic models might be significantly enhanced by incorporating densitometric parameters and machine learning techniques.

## Introduction

Pediatric traumatic brain injury (TBI) accounts for over 500,000 emergency department visits in the United States each year (1). The anatomical and physiological properties (e.g., thinner cranium, less myelinated tissue) of the pediatric brain can result in more rapid and severe development of secondary ischemic-edematous insults after head injury (2–4), and hence worse outcomes, than in adults (5). Given that cerebral edema is the fundamental pathophysiological mechanism underlying TBI, assessing the extent of cerebral edema may be crucial for evaluating the risk of intracranial hypertension and predicting outcomes (6).

Magnetic resonance imaging (MRI) is of significant diagnostic value for identifying pathological diffuse brain swelling; however, it requires children to be stationary and often sedated during a long acquisition time. Therefore, brain computed tomography (CT) remains the gold standard imaging modality during the acute phase of TBI for rapidly evaluating TBI and developing an appropriate intervention strategy (7, 8). The degree of brain swelling is generally evaluated by CT classification systems [i.e., Marshall (9) and Rotterdam score (10)] based on the status of the mesencephalic cisterns or midline shift; this classification is easily applicable and is considered useful for predicting outcomes after TBI (9–12). The efficacy of CT classification systems makes them important prognostic factors in well-known TBI prognostic models (i.e., Corticosteroid Randomization after Significant Head Injury [CRASH] or International Mission for Prognosis and Analysis of Clinical Trials [IMPACT] models) (13, 14). However, the classification system relies on manual visual inspection of CT images by clinicians and hence has been criticized for its intrinsic inter- and intrarater variabilities (10, 15).

It is well-known that there is a linear relationship between edema-induced water accumulation and Hounsfield unit (HU) values (16); a 1% increase in tissue water content causes a 2-3 HU reduction in attenuation on CT images (17). This dose-responsive relationship could be applied to quantitatively evaluate edematous changes by using HU distribution via densitometric CT analysis in patients with TBI (18). Accordingly, densitometric analysis could be an appropriate tool for evaluating the majority of early-stage pediatric TBIs that show no visually identifiable abnormalities on CT images. Moreover, by performing whole-cerebrum densitometric analysis in a fully automated manner, the degree of secondary edematous accumulation following TBI could be objectively measured without inter- or intrarater variability (18). These advantages of densitometric CT analysis could be exploited to construct a more robust prognostic model for TBI.

The past few years have seen a surge in attempts to use machine learning to construct prognostic models. Traditional logistic regression may have low robustness for explaining multivariate non-linear relationships (19), whereas machine learning can better apprehend non-linear relationships and interactions through more flexible modeling (20). Gradient-boosted decision trees (GBDTs), a widely used machine learning algorithm, produce an interpretable prognostic model that is an ensemble of decision trees, which are in high demand in the medical domain (21). Among GBDT variants, CatBoost has recently been introduced and has shown notable robustness and highly accurate generalizability (22). This study hypothesized that combining CT densitometry and a machine learning technique (i.e., CatBoost) would enhance the prognostic value of brain CT with a more robust prognostic model for pediatric TBI. The objectives of this study are 2-fold: (1) to investigate the association between intracranial densitometry based on brain CT and various outcomes in pediatric TBI patients and (2) to evaluate the prognostic value of brain CT by constructing a densitometry-augmented TBI prognostic model based on a robust machine learning method.

## Materials and Methods

### Study Design and Setting

**Figure 1:**
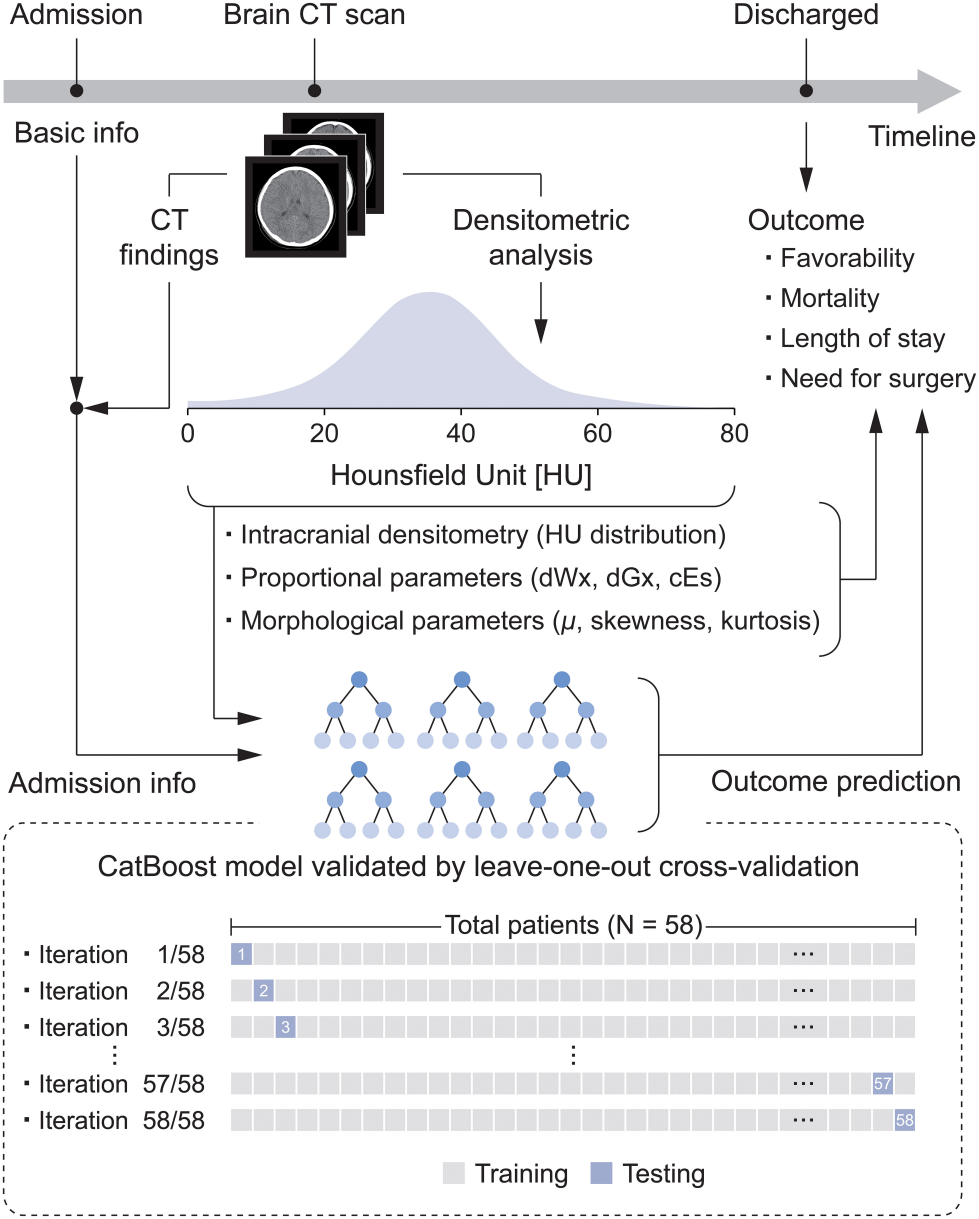
Flow diagram for the development of the densitometry-augmented prognostic model based on brain CT.

This retrospective pilot study investigated the relationship between intracranial densitometry based on brain CT and the outcome of pediatric patients with TBI. Furthermore, TBI prognostic models were constructed using the CatBoost model (22), a cutting-edge gradient boosting algorithm optimized in a small dataset and robust to overfitting, based on intracranial densitometric information. The prognostic models were evaluated through leave-one-out cross-validation (LOOCV), which is appropriate for small datasets. An overview of this study is shown in Figure 1.

The setting of this study is at a single trauma center (i.e., the Trauma Center of Seoul National University Hospital). Basic clinical information of TBI patients, such as brain CT images and Glasgow Coma Scale (GCS) scores, was obtained during the same period. Anonymized clinical information obtained on admission to the emergency room, radiology reports, and brain CT images were retrospectively reviewed and collected from the institutional database of Seoul National University Hospital from 2013 to 2017. This study was approved by the Ethics Committee of Seoul National University Hospital (IRB H-1706-144-862). The requirement for informed consent was waived due to the retrospective nature of this study.

### Study Population

The subjects were enrolled according to the following inclusion criteria: (a) direct admission for TBI through the emergency medicine department (not transferred from another hospital); (b) age ≤ 19 years; (c) underwent non-enhanced brain CT scan; and (d) eligible CT acquisition conditions (tube voltage = 120 kVp, tube current ≥ 150 mA) for analysis of HU values (23). Additionally, the following exclusion criteria were applied: (a) significant image artifacts (e.g., beam-hardening effects) in the CT images and (b) outcome unrelated to traumatic head injury (e.g., acute respiratory failure, myocardial contusions). Furthermore, patients with significant infratentorial hemorrhage were excluded because even a small infratentorial lesion may be fatal, which would significantly affect the outcomes and skew the data (24).

These criteria were entered into the institutional clinical data warehouse of Seoul National University Hospital, SUPREME®. Consequently, a total of 58 pediatric TBI patients who underwent head CT examinations were retrospectively included in this pilot study. Enrolled subjects were directly transferred to the trauma center. The Glasgow Outcome Scale (GOS) score was recorded at the time of discharge. Additionally, alternative outcome measures such as in-hospital mortality, length of stay (LOS) and need for surgery were used as reference standards.

### Densitometric Analysis of Brain CT

Densitometric CT analysis utilizes HU values obtained by brain CT to derive intracranial densitometric data as a distribution representing the material density. It can provide a quantitative evaluation of brain density alterations caused by edema-induced water accumulation without inter- or intrarater variability. This study utilized the methods proposed by Kim et al. (18), which allow the quantitative derivation of intracranial densitometric data of the whole cerebrum. To implement the method, in-house software was written in Java (Oracle, Inc., Redwood Shores, California, USA), providing a fully automatic method for the densitometric analysis of head CT images. To analyze only the major intracranial components (e.g., cerebrospinal fluid, parenchyma, blood), a threshold limit of 0–79 HU was applied (18). The intracranial densitometry was derived from Equation 1:


(1) $$\begin{array}{l} p\left(\lambda \right)=\frac{\sum_{k\;=\;1}^{n}\lambda_{k}}{\sum_{k\;=\;1}^{n}\sum_{\lambda \;=\;0}^{79}\lambda_{k}} \end{array}$$

where λ_*k*_ is the number of pixels having an HU value of λ from the *k*^th^ CT image in a series of CT scans showing the supratentorial brain region, the denominator of the equation is the entire number of pixels having an HU value of 0 to 79 in the whole cerebrum, and *p*(λ) is the proportion of pixels having an HU value of λ in the whole cerebrum. The graph of the intracranial densitometric data was derived by plotting the *p*(λ) in the range of 0–79 HU.

In this study, densitometric analysis was based on initial brain CT scans at admission and follow-up CT scans acquired within 48 h of initial CT scans. Brain CT scans were obtained with a Brilliance 64 scanner (Philips Medical Systems, Eindhoven, Netherlands). The CT acquisition parameters were as follows: tube voltage = 120 kVp, tube current ≥ 150 mA, and image matrix = 512 by 512, with a 5-mm slice thickness. Follow-up CT scans were also performed with the same acquisition settings.

### Quantitative Evaluation of Intracranial Densitometry

There are two paradigms to quantitatively evaluate intracranial densitometry: one for evaluating the proportion of a specific HU region and the other for evaluating the morphology of the HU distribution.

In evaluating the proportion of a specific HU region, three parameters, dWx, dGx, and cEs, proposed in previous studies were used in the study (18, 25). Excessive water accumulation caused by cerebral edema or ischemia lowers the material density of the brain parenchyma and consequently affects both white matter (WM) and gray matter (GM). In addition, the presence of space-occupied lesions with high density (e.g., subdural, epidural, and intracerebral hemorrhage) also decreased the relative proportion of normal density parenchyma in the cranium. Accordingly, the proportions of normal-density WM (dWx) and GM (dGx) were quantitatively assessed. dWx and dGx were defined as the proportions of pixels of 26–30 and 31–35 HU, respectively, among pixels depicting the entire cerebrum (25). In addition, Kim et al. suggested the use of the cerebral edema score (cEs), ranging from 17 to 24 HU, as an indicator of cerebral edema severity to compensate for the CT classification system (18).

The presence of a hypodense lesion (e.g., ischemic-edematous lesion) or hyperdense lesions (e.g., intracranial hemorrhage) in the intracranial area can contribute to the left- and right-sided dominance of the densitometry, respectively. Such pathological brain changes can be identified by assessing the intracranial densitometric morphology, which was evaluated as the proportional HU distribution by calculating μ, skewness and kurtosis, where μ is the mean HU value of the distribution, and skewness and kurtosis are the measure of the asymmetry and the tail of the distribution, respectively.

**Figure 2:**
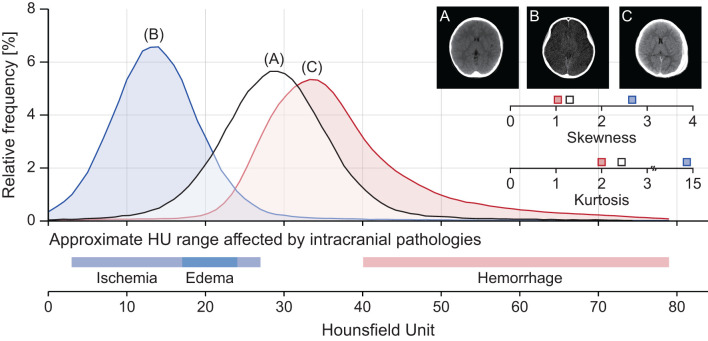
Representative cases of intracranial densitometry affected by various TBI pathologies. **(A)** A 7-year-old male patient with no visible pathology, **(B)** A 10-year-old female patient with massive brain edema, **(C)** A 7-year-old male patient with acute subdural hemorrhage. The color bar at the bottom shows the HU range on densitometry, which mainly affects intracranial pathologies.

Intracranial densitometry reveals pathological changes in the material density of the whole cerebrum. Thus, it may be suggestive of various TBI pathologies (e.g., cerebral ischemia, edema, intracranial hemorrhage). Figure 2 shows some intuitive examples of intracranial densitometry affected by various TBI pathologies. The color bar at the bottom shows the HU area on densitometry, where cerebral ischemia [3–27 HU] (26), edema [17–24 HU] (18), and intracranial hemorrhage [40 HU~] (27) are primarily affected. Compared with the patient without significant visible pathology (Figure 2A), the patient with massive brain edema following severe ischemic insults (Figure 2B) showed a leftward shift of the densitometry center with an increase in both skewness and kurtosis due to excessive water accumulation. On the other hand, in the patient with a large degree of acute subdural hemorrhage (Figure 2C), the center of the densitometry shifted to the right due to the space-occupied lesion. In addition, both Figures 2B,C show that the proportion of parenchyma within the normal density range was also affected by the occurrence of intracranial pathology.

Lesions with partially overlapping HU ranges (e.g., subacute subdural hemorrhage) may skew the interpretation of the densitometric parameters, but only patients during the acute stage were involved in this study.

### Prognostic Model Construction

Prognostic models were constructed based on the four outcome measures (i.e., favorability, mortality, LOS and need for surgery) with CatBoost (22), which is a state-of-the-art gradient-boosted decision tree. CatBoost mitigates the overfitting problem and shows robustness and generalizability. In addition, CatBoost has various advantages of (1) having a swift training speed through parallel processing, (2) being appropriate for small sample sizes and unbalanced data, and (3) exhibiting higher accuracy than other gradient boosting algorithms. The IMPACT model was excluded from this study because the IMPACT model does not consider patients under the age of 14 years (14).

In this study, a total of three types of prognostic models were constructed. (1) CatBoost-based CRASH-CT (i.e., CRASH-CT_CatBoost_) is the model based on admission characteristics (i.e., age, GCS, pupil reaction, and presence of extracranial injury) and initial CT findings (i.e., presence of petechial hemorrhage, obliteration of the third ventricle or basal cisterns, subarachnoid hemorrhage, midline shift, and non-evacuated hematoma). These are the same input of the conventional CRASH-CT model (13). (2) The densitometry-augmented model (i.e., D/CRASH-CT_CatBoost_) was constructed with the intracranial densitometric information (i.e., dWx, dGx, cEs, μ, skewness, kurtosis, and HU distribution) and the CRASH-CT input. (3) The reference model was the conventional CRASH-CT model (i.e., CRASH-CT_LR_) established by logistic regression.

Prognostic models for outcome favorability were derived from both initial and follow-up CT to assess the changes in prognostic value during the acute phase of TBI. Undoubtedly, the model based on the follow-up CT data uses input variables derived from the follow-up CT. On the other hand, prognostic models for mortality, LOS, and need for surgery were all established only based on initial CT data at admission.

The prognostic value of CatBoost was optimized by a grid search with the variation of three hyperparameters: Iteration [50, 100, 200, 300, 400], depth [4, 5, 6, 7, 8, 9, 10], and loss function [log loss, cross-entropy]. Likewise, the logistic regression model was optimized with iterations [50, 100, 200, 300, 400] and C [0.001, 0.01, 0.1, 1, 10, 100] as hyperparameters.

Principal component analysis (PCA) was used to reduce the dimensions of the input variables that still contain most of the information to alleviate the dimensionality and overfitting problems that can occur in machine learning procedures. Accordingly, the high dimensions of admission characteristics, initial CT findings, and HU distribution were reduced by PCA.

SHAPs (Shapley Additional exPlanations) of the tree ensemble model were derived to assess the importance of the model input variables. Based on this, it is possible to conveniently examine the importance of individual input variables used in the CatBoost-based prognostic model and to determine how the input variables contribute to the prediction of the outcomes.

The predictive performance of all prognostic models was assessed by LOOCV (Figure 1), which results in unbiased and reliable estimates of model performance; iterative validations were performed 58 times by separating training (*N* = 57) and testing subjects (*N* = 1). These machine learning models were developed by scikit-learn 0.24.1 and CatBoost library 0.25.1 in the Python 3.7 environment.

### Statistical Analysis

The calculation of the sample size was performed by Viechtbauer's formula developed for the pilot study (28); the calculated sample size was 45, with a confidence level of 0.90 and a probability of 0.05. Considering that the dropout rate was 30%, 58 subjects were enrolled in this study. Non-parametric statistical methods were employed due to the sample size. The Mann-Whitney *U*-test was applied to compare continuous data between outcome groups. The discrimination of the prognostic models was assessed by receiver operating characteristic (ROC) curve analysis. The optimal cutoff values for discriminating outcomes were calculated using the maximal Youden's J statistic (sensitivity + specificity – 1) (29) in the ROC curve. Hanley's test (30) was performed to compare the area under the ROC curve (AUC). In this study, four reference standards against AUC of all prognostic models were used: (1) dichotomous outcome measured at discharge [i.e., GOS score 1–3 (positive) vs. 4–5 (negative)], (2) in-hospital mortality [i.e., deceased (positive) vs. survived (negative)], (3) LOS in hospital [i.e., > 1 week (positive) vs. ≤ 1 week (negative)], and (4) need for surgical intervention [i.e., underwent TBI-related surgery during hospitalization (positive) vs. no need for surgical procedure (negative)]. In this study, there was no need to reassign the LOS outcome of the deceased patients because no patients died in the group with LOS of <1 week (31). The sensitivity, specificity, positive predictive value, and negative predictive value with 95% confidence interval of each prognostic model were determined at the optimal cutoff. The analyses were considered statistically significant at two-sided *p* < 0.05. Statistical analyses were conducted using commercial software (SPSS 24, IBM Corp., Chicago, Illinois, USA).

## Results

### Demographics

Fifty-eight pediatric TBI patients were included in this study. Of the 58 patients, 46 (79.3%) were assigned to the favorable outcome group, and 12 (20.7%) were assigned to the unfavorable outcome group. The detailed demographics are listed in Table 1.

**Table 1:** Baseline characteristics.

	**Total** **(*N* = 58)**	**Favorable outcome group** **(*N* = 46)**	**Unfavorable outcome group** **(*N* = 12)**
**Age, years**			
Median, interquartile range	6 (1.75–13)	7 (2–14.25)	5 (1–9.25)
**Sex, no. (%)**			
Male	32 (55.2)	27 (58.7)	5 (41.7)
Female	26 (44.8)	19 (41.3)	7 (58.3)
**Cause of injury, no. (%)**			
Motor vehicle accident	17 (29.3)	15 (32.6)	2 (16.7)
Fall	24 (41.4)	22 (47.8)	2 (16.7)
Blunt trauma	17 (29.3)	9 (19.6)	8 (66.7)
**Glasgow Coma Scale on admission, no. (%)**			
3–8	9 (15.5)	5 (10.9)	4 (33.3)
9–12	3 (5.2)	2 (4.3)	1 (8.3)
13–15	46 (79.3)	39 (84.8)	7 (58.3)
**Pupil reactivity, no. (%)**			
Both	53 (91.4)	42 (91.3)	11 (91.7)
One	3 (5.2)	3 (6.5)	0 (0)
None	2 (3.4)	1 (2.2)	1 (8.3)
**Extracranial injury, no. (%)**			
Extracranial hematoma	7 (12.1)	6 (13.0)	1 (8.3)
Facial injury	8 (13.8)	7 (15.2)	1 (8.3)
Lower extremity injury	4 (6.9)	4 (8.7)	0 (0)
Spinal cord injury	3 (5.2)	3 (6.5)	0 (0)
None	36 (62.1)	26 (56.5)	10 (83.3)
**Surgical intervention, no. (%)**			
Burr hole trephination	4 (6.9)	4 (8.7)	0 (0)
Craniectomy/Craniotomy	9 (15.5)	7 (15.2)	2 (16.7)
External ventricular drain	1 (1.7)	0 (0)	1 (8.3)
None	44 (75.9)	35 (76.1)	9 (75.0)
**Elapsed time between initial CT acquisition and trauma, hours**			
Median, interquartile range	11.50 (4.38–27.79)	10.09 (4.00–26.33)	12.50 (6.88–65.37)
Follow-up CT scan within 48 h, no. (%)	34 (58.6)	28 (60.9)	6 (50.0)
**Imaging findings, no. (%)**			
Skull fracture	18 (31.0)	16 (34.8)	2 (16.7)
Petechial hemorrhage	10 (17.2)	8 (17.4)	2 (16.7)
Obliteration of basal cisterns	9 (15.5)	7 (15.2)	2 (16.7)
Midline shift > 5 mm	25 (43.1)	19 (41.3)	6 (50.0)
**Length of stay, days**			
Median, interquartile range	9 (3–25)	6 (2.75–16.5)	20.5 (12.5–153.75)
In-hospital mortality, no. (%)	6 (10.3)	0 (0)	6 (50.0)

### Changes in Intracranial Densitometry at the Acute Phase of Pediatric TBI

**Figure 3:**
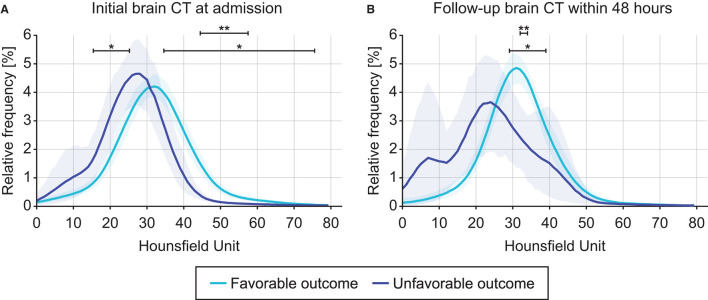
Comparison of intracranial densitometry by outcome favorability based on the initial brain CT at admission **(A)** and follow-up brain CT within 48 h **(B)**. Blue = favorable outcome group, purple = unfavorable outcome group. The bold line and shaded area denote the mean and range of the 95% confidence interval, respectively. Significance at *p* < 0.05* and *p* < 0.001**.

For initial CT acquired at admission and follow-up CT acquired within 48 h later, the changes in intracranial densitometry according to the outcome were evaluated. Figure 3A shows the density distribution obtained from the initial CT scan at admission, which skewed to the right in the unfavorable outcome group compared to the density distribution of the favorable outcome group, indicating brain density alteration in the acute phase of TBI. These morphological disagreements resulted in significant differences between the outcome groups in the specific HU range. Contrary to the intracranial densitometry of the favorable outcome relatively analogous to the normal distribution, the skewed distribution of the unfavorable outcome became more distinguishable in the follow-up CT (Figure 3B). This deformation suggests that the change in brain density due to secondary insults became more substantial.

The intracranial densitometric morphology was further assessed through densitometric parameters in a quantitative manner. Table 2 shows the densitometric parameters in both outcome groups. Among the proportional densitometric parameters from the initial CT, cEs showed a significant difference, whereas dWx and dGx, indicating the proportion of normal-density WM and GM, showed no significance. Nevertheless, only the dGx from the follow-up CT showed a significant difference. As the intracranial morphology of the unfavorable outcome group showed a distorted distribution, all of the morphological densitometric parameters differed significantly between outcome groups both in initial and follow-up CT. On the other hand, the CT classification systems (i.e., Marshall and Rotterdam score) did not significantly distinguish outcome favorability in either initial or follow-up CT.

**Table 2:** Comparison of densitometric parameters and conventional CT classification systems by outcome favorability based on the initial and follow-up brain CT.

	**Median (interquartile range)**		***P*-value**
	**Favorable outcome**	**Unfavorable outcome**	
**Initial brain CT at admission (N = 58; favorable = 46, unfavorable = 12)**			
**Proportional densitometric parameters**			
dWx	18.94 (13.43–25.17)	24.00 (15.31–27.90)	0.19
dGx	20.48 (14.92–24.48)	18.87 (7.08–22.07)	0.28
cEs	12.40 (7.15–19.40)	24.26 (15.58–39.71)	0.008
**Morphological densitometric parameters**			
μ	32.89 (27.73–35.78)	28.32 (22.39–29.51)	0.005
Skewness	0.55 (0.09–1.17)	1.43 (0.76–1.73)	0.006
Kurtosis	2.28 (1.15–4.74)	4.46 (4.08–7.79)	0.002
**CT classification systems**			
Marshall score	6 (2.75–6)	4 (2.50–6)	0.33
Rotterdam score	2 (2-2)	2 (1.25–2.75)	0.97
**Follow-up brain CT within 48 h (*****N*** **= 34; favorable = 28, unfavorable = 6)**			
**Proportional densitometric parameters**			
dWx	22.71 (15.15–27.59)	14.88 (6.75–26.07)	0.11
dGx	23.45 (19.99–26.23)	13.03 (2.63–18.50)	0.001
cEs	13.12 (8.35–19.00)	31.38 (5.09–40.09)	0.30
**Morphological densitometric parameters**			
μ	31.65 (29.14–34.26)	26.67 (16.20–30.11)	0.037
Skewness	0.69 (0.06–1.07)	1.57 (0.69–1.86)	0.022
Kurtosis	3.05 (1.56–4.39)	5.01 (3.89–8.61)	0.015
**CT classification systems**			
Marshall score	5.5 (4–6)	5 (3.75–6)	0.95
Rotterdam score	2 (1.25–3)	2.5 (2–3)	0.39

### Prognostic Model at the Acute Phase of Pediatric TBI Augmented by Intracranial Densitometry

**Figure 4:**
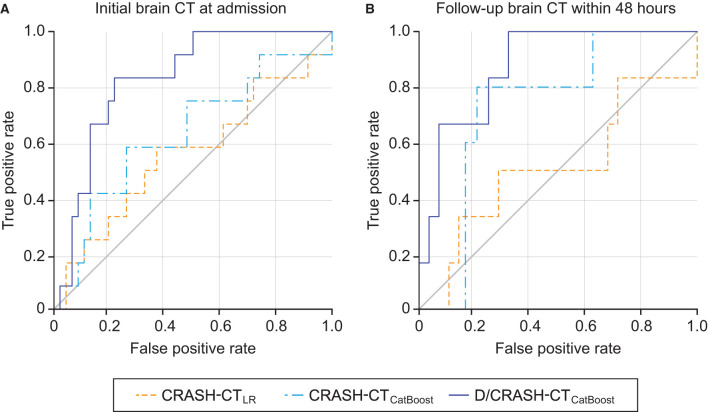
ROC curves of the prognostic models for the differentiation of favorable and unfavorable outcomes based on the initial brain CT at admission **(A)** and follow-up brain CT within 48 h **(B)** by the LOOCV. Orange = CRASH-CT_LR_, blue = CRASH-CT_CatBoost_, indigo = D/CRASH-CT_CatBoost_.

The predictive performance of the prognostic models constructed from the variables acquired during the acute phase was assessed by LOOCV based on the outcome favorability (Figure 4). As the reference model, the CRASH-CT model based on logistic regression (i.e., CRASH-CT_LR_) showed an AUC of 0.56. By using the CatBoost model, an AUC of 0.63 was derived from the model using only the CRASH-CT input (i.e., CRASH-CT_CatBoost_). The densitometry-augmented model (i.e., D/CRASH-CT_CatBoost_) showed significant enhancement in the AUC compared with the CRASH-CT_LR_ model. Specifically, the AUC of the model that included densitometric information was improved to 0.83 (*p* < 0.03 by Hanley's test). Alternating the variables from the initial CT to the follow-up CT further enhanced the prognostic value of the CatBoot models (Figure 4B). Accordingly, the difference in the AUC between the CRASH-CT_LR_ and the D/CRASH-CT_CatBoost_ became more significant (*p* < 0.02 by Hanley's test) despite the smaller number of subjects. The performance of the prognostic models at the optimal cutoff point for predicting the outcome was further evaluated (Table 3).

**Table 3:** Comparison of prognostic models for predicting outcome favorability based on the initial and follow-up CT-based LOOCV.

	**AUC**	**Sensitivity [%]**	**Specificity [%]**	**PPV [%]**	**NPV [%]**
**Initial brain CT at admission (*****N*** **= 58)**					
CRASH-CT_LR_	0.56 (0.36–0.76)	58.33 (31.95–80.67)	63.04 (48.60–75.48)	29.17 (14.91–49.17)	85.29 (69.87–93.55)
CRASH-CT_CatBoost_	0.63 (0.44–0.83)	58.33 (31.95–80.67)	73.91 (59.74–84.4)	36.84 (19.15–58.96)	87.18 (73.29–94.4)
D/CRASH-CT_CatBoost_	0.83 (0.72–0.94)	83.33 (55.20–95.30)	78.26 (64.43–87.74)	50.00 (29.93–70.07)	94.74 (82.71–98.54)
**Follow-up brain CT within 48 h (*****N*** **= 34)**					
CRASH-CT_LR_	0.51 (0.21–0.82)	50.00 (18.76–81.24)	71.43 (52.94–84.75)	27.27 (9.75–56.56)	86.96 (67.87–95.46)
CRASH-CT_CatBoost_	0.73 (0.51–0.96)	80.00 (37.55–96.38)	79.17 (59.53–90.76)	44.44 (18.88–73.33)	95.00 (76.39–99.11)
D/CRASH-CT_CatBoost_	0.88 (0.74–1.00)	83.33 (43.65–96.99)	75.00 (56.64–87.32)	41.67 (19.33–68.05)	95.45 (78.20–99.19)

*AUC, area under the ROC curve; PPV, positive predictive value; NPV, negative predictive value*.

**Figure 5:**
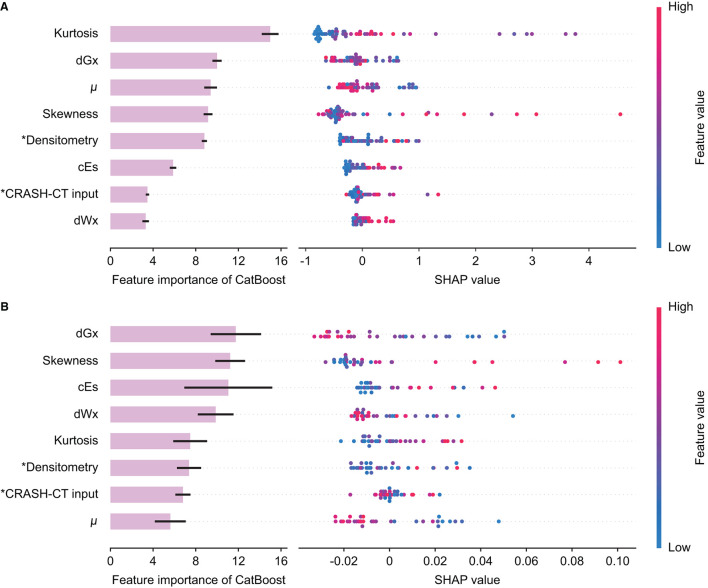
Feature importance and SHAP value of the D/CRASH-CT_CatBoost_ model for the differentiation of favorable and unfavorable outcomes based on the initial brain CT at admission **(A)** and follow-up brain CT within 48 h **(B)**. Each row shows the distribution of SHAP values assigned to a model input. Asterisks denote input variables that have undergone dimensionality reduction by principal component analysis.

The feature importance of the input variable and SHAP of the tree ensemble of the best model from the initial CT, D/CRASH-CT_CatBoost_, was derived (Figure 5A). In the D/CRASH-CT_CatBoost_ model, morphological parameters (i.e., kurtosis, skewness, and μ) and dGx were the main contributors to the prediction. As kurtosis and skewness increased, the prediction of unfavorable outcomes increased. Additionally, lower μ and dGx values contributed to the prediction of unfavorable outcomes. D/CRASH-CT_CatBoost_ from the follow-up CT also showed that dGx and skewness mainly contributed to the outcome prediction (Figure 5B), which is similar to the model from the initial CT. This may reflect the shift in the center of intracranial densitometry toward lower HUs with morphological distortion in patients with an unfavorable outcome.

### Comparative Assessment of Intracranial Densitometry According to Alternative TBI Outcome Measures

**Figure 6:**
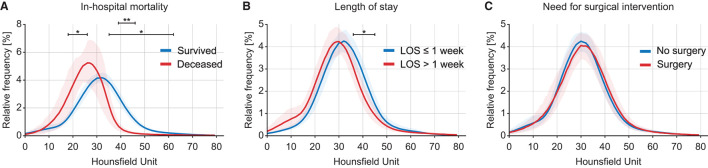
Comparison of intracranial densitometry by alternative outcome measures: in-hospital mortality **(A)**, length of stay **(B)** and need for surgical intervention **(C)**. The bold line and shaded area denote the mean and range of the 95% confidence interval, respectively. Significance at *p* < 0.05* and *p* < 0.001**.

Intracranial densitometry acquired at admission was further compared for alternative outcome measures for TBI (i.e., in-hospital mortality, LOS, and need for surgical intervention). Intracranial densitometry in the deceased group showed considerable skewness, suggesting that low-attenuated pixels dominate (Figure 6A). On the other hand, when the groups were dichotomized based on the LOS (1 week), the intracranial densitometry of the worse outcome group was transversely shifted to the left without noticeable deformation (Figure 6B). However, there was no association between need for surgery and intracranial densitometry; the morphology between the outcome groups (i.e., need for surgery vs. no surgery) was indistinguishable (Figure 6C). In addition, Table 4 further describes the densitometric parameters and CT classifications that depend on the three outcome measures. Outcome groups based on in-hospital mortality and LOS revealed significant morphological parameters, although proportional parameters and CT scores showed limited significance.

**Table 4:** Comparison of densitometric parameters and conventional CT classification systems by alternative outcome measures.

	**In-hospital mortality**			**Length of stay**			**Need for surgical intervention**		
	**Median (interquartile range)**		***P*-value**	**Median (interquartile range)**		***P*-value**	**Median (interquartile range)**		***P*-value**
	**Survival** **(*N* = 52)**	**Deceased** **(*N* = 6)**		**LOS ≤ 1 week** **(*N* = 28)**	**LOS > 1 week** **(*N* = 30)**		**No need for surgery** **(*N* = 44)**	**Need for TBI-related surgery** **(*N* = 14)**	
**Proportional densitometric parameters**									
dWx	19.27 (13.64–25.22)	26.22 (13.48–34.85)	0.19	18.84 (14.24–24.28)	21.86 (11.92–27.42)	0.52	19.48 (14.53–27.09)	19.93 (13.04–24.33)	0.57
dGx	20.32 (14.82–24.23)	18.38 (3.14–24.21)	0.32	20.48 (16.61–24.55)	18.90 (13.79–23.00)	0.37	20.03 (13.78–24.85)	20.51 (17.68–22.99)	0.81
cEs	13.25 (7.47–20.72)	26.87 (20.64–45.44)	0.011	10.90 (7.29–18.89)	17.55 (9.11–27.72)	0.13	14.71 (7.54–26.53)	15.14 (8.40–22.52)	0.96
**Morphological densitometric parameters**									
μ	32.15 (27.31–35.64)	27.51 (20.43–29.36)	0.022	33.25 (28.49–35.78)	29.24 (26.41–33.72)	0.06	30.47 (26.66–34.81)	31.16 (27.04–35.62)	0.61
Skewness	0.57 (0.10–1.18)	1.67 (0.91–1.78)	0.007	0.4 (−0.05 to 0.98)	1.08 (0.46–1.57)	0.003	0.89 (0.15–1.28)	0.45 (0.10–1.04)	0.37
Kurtosis	2.45 (1.22–4.66)	7.14 (4.49–9.63)	0.003	2.31 (1.12–4.06)	4.10 (1.77–6.02)	0.07	3.39 (1.50–5.41)	1.96 (1.12–4.07)	0.17
**CT classification systems**									
Marshall score	6 (3–6)	3 (2–4.5)	0.034	6 (2.25–6)	6 (2.75–6)	0.76	6 (2–6)	6 (4–6)	0.50
Rotterdam score	2 (2)	1.5 (1–2.25)	0.20	2 (1.25–2)	2 (2)	0.75	2 (1.25–2)	2 (2–3)	0.10

### Prognostic Model for Alternative TBI Outcome Measures Augmented by Intracranial Densitometry

**Figure 7:**
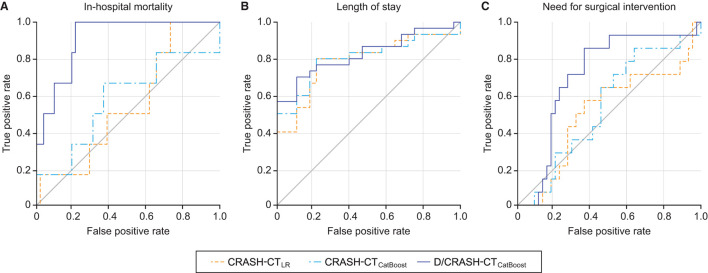
ROC curves of the prognostic models for the differentiation of alternative outcome measures: in-hospital mortality **(A)**, length of stay **(B)** and need for surgical intervention **(C)** based on LOOCV. Orange = CRASH-CT_LR_, blue = CRASH-CT_CatBoost_, indigo = D/CRASH-CT_CatBoost_.

For the three alternative outcome measures, the performance of the prognostic models was evaluated through LOOCV (Figure 7). Figure 7A shows exceptionally superior performance of the D/CRASH-CT_CatBoost_ than that of the CRASH-CT_LR_ for predicting in-hospital mortality (*p* < 0.018 by Hanley's test). On the other hand, prognostic models for LOS prediction showed consistent capacity without a significant difference between the prognostic models (Figure 7B). The prognostic ability of the model to predict the need for surgery showed a moderate AUC of 0.71. Table 5 indicates detailed statistical measures for the performance of the prognostic model.

**Table 5:** Comparison of prognostic models for predicting alternative outcome measures based on LOOCV.

	**AUC**	**Sensitivity [%]**	**Specificity [%]**	**PPV [%]**	**NPV [%]**
**In-hospital mortality**					
CRASH-CT_LR_	0.55 (0.32–0.79)	50.00 (18.76–81.24)	61.54 (47.96–73.53)	13.04 (4.54–32.13)	91.43 (77.62–97.04)
CRASH-CT_CatBoost_	0.58 (0.29–0.87)	66.67 (30–90.32)	63.46 (49.87–75.2)	17.39 (6.98–37.14)	94.29 (81.39–98.42)
D/CRASH-CT_CatBoost_	0.91 (0.82–1.00)	100 (60.97–100)	78.85 (65.97–87.76)	35.29 (17.31–58.70)	100 (91.43–100)
**Length of stay**					
CRASH-CT_LR_	0.79 (0.67–0.91)	80.00 (62.69–90.49)	78.57 (60.46–89.79)	80.00 (62.69–90.49)	78.57 (60.46–89.79)
CRASH-CT_CatBoost_	0.80 (0.68–0.92)	80.00 (62.69–90.49)	78.57 (60.46–89.79)	80.00 (62.69–90.49)	78.57 (60.46–89.79)
D/CRASH-CT_CatBoost_	0.83 (0.72–0.94)	76.67 (59.07–88.21)	78.57 (60.46–89.79)	79.31 (61.61–90.15)	75.86 (57.89–87.78)
**Need for surgical intervention**					
CRASH-CT_LR_	0.51 (0.33–0.70)	57.14 (32.59–78.62)	63.64 (48.87–76.22)	33.33 (17.97–53.29)	82.35 (66.49–91.65)
CRASH-CT_CatBoost_	0.54 (0.37–0.71)	64.29 (38.76–83.66)	54.55 (40.07–68.29)	31.03 (17.28–49.23)	82.76 (65.45–92.40)
D/CRASH-CT_CatBoost_	0.71 (0.56–0.86)	85.71 (60.06–95.99)	63.64 (48.87–76.22)	42.86 (26.51–60.93)	93.33 (78.68–98.15)

*AUC, area under the ROC curve; PPV, positive predictive value; NPV, negative predictive value*.

**Figure 8:**
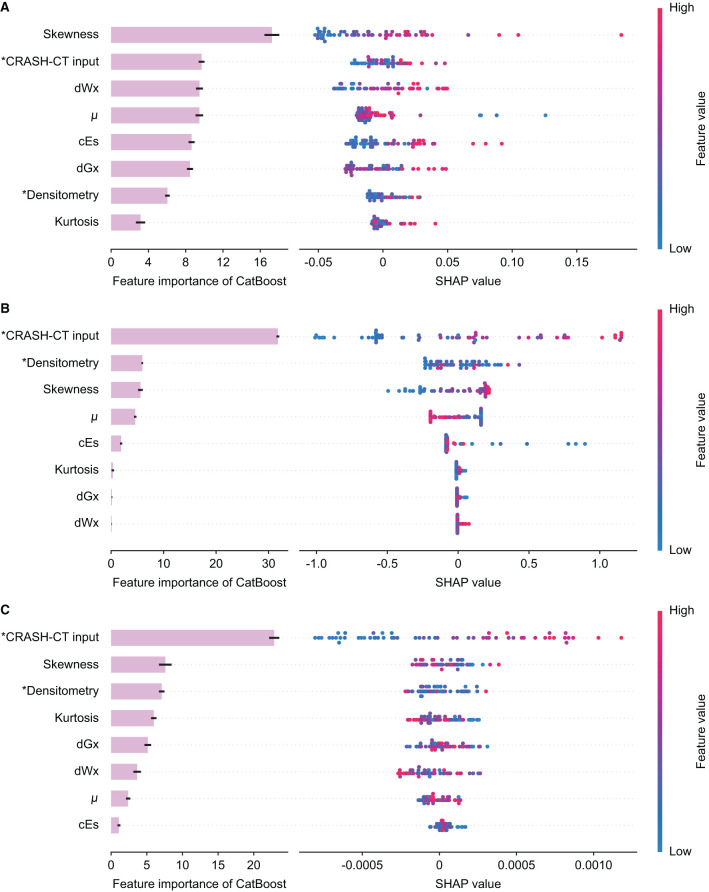
Feature importance and SHAP value of the D/CRASH-CT_CatBoost_ model for the differentiation of alternative outcome measures: in-hospital mortality **(A)**, length of stay **(B)**, and need for surgical intervention **(C)**. Each row shows the distribution of SHAP values assigned to a model input. Asterisks denote input variables that have undergone dimensionality reduction by principal component analysis.

The feature importance and SHAP of the tree ensemble of the D/CRASH-CT_CatBoost_ were derived based on the three outcome measures (Figure 8). The in-hospital mortality prediction model possessed the skewness of intracranial densitometry as a significant contributor, but in the models predicting LOS or need for surgery, the conventional CRASH-CT input, not the densitometric parameter, made the most dominant contribution to the prediction. This result was related to the weak statistical significance of the input variables from the intracranial densitometry.

## Discussion

The efficacy and practicality of the conventional CT classification system for evaluating injury severity after TBI have long been acknowledged; however, this method suffers from high inter- and intrarater variability (10, 15). Non-etheless, the system has been incorporated into well-known prognostic models for TBI, which may significantly affect the robustness of the models. This study utilized a recently developed interpretable machine learning model (CatBoost) to build a TBI prognostic model and employed CT densitometry, which is fully automated and thus does not suffer from inter- and intrarater variability (18), to compensate for the subjective CT classification system. The results indicate that (1) densitometric parameters are independently associated with various TBI outcome measures (i.e., favorability, mortality, and LOS) but not the need for surgery, (2) the prognostic capacity of the conventional CRASH model could be enhanced by employing CatBoost rather than traditional logistic regression, and (3) the capacity can be further increased by supplementing densitometric parameters as model inputs. The novelty and importance of the utilized methods and derived findings of this study warrant a detailed discussion.

### The Rationale of Densitometric CT Analysis in TBI Pathologies

The inter- and intrarater variability of conventional CT classification systems mainly stems from the wide heterogeneity of injury types and severity (15) and the dependence on arbitrary estimations based on visual inspection (10). Densitometric CT analysis aims to overcome such limitations by quantitatively evaluating the density of brain structures and/or lesions based on brain CT scans. The technique has been widely applied in evaluations of various pathological brain changes [e.g., early cerebral edema (18, 32) or ischemic changes (33), lesion water uptake (34), parenchymal compression (35), and hemorrhage growth (36)] and for predicting outcomes (18, 25, 37, 38) in acquired brain injury. There are two approaches to applying densitometry: region of interest-based analysis (33–36, 38) and whole-cerebrum analysis (18, 25, 37). This study adopted the latter approach (1) to ensure the robustness of the prognostic model to be constructed and (2) because it was originally designed to be applied for pediatric TBI (18).

Whole-cerebrum analysis-based intracranial densitometry can respond to two major intracranial pathologies of TBI: ischemic-edematous insults and intracranial hemorrhage. However, there is a discrepancy in the sensitivity of intracranial densitometry for the two pathologies. According to volumetric CT measurements, the proportion of parenchyma in pediatric subjects accounts for 93–94% of the cranium (39). Undoubtedly, parenchyma also occupies the equivalent proportion in intracranial densitometry. Therefore, intracranial densitometry derived from whole-cerebrum analysis benefits from the sensitive response to diffuse ischemic injury that contributes to global changes in parenchymal density, despite subtle alterations (18, 25). Of course, the significant amount of hyperdense lesion (e.g., crescent-shaped acute subdural hemorrhage) also markedly expanded the proportion of the intracranial densitometry area above 40 HU (Figure 2C). On the other hand, whole-cerebrum densitometry shows a limited response to focal lesions occupying a relatively small proportion. For example, intracranial densitometry may be less sensitive for focal lesions such as petechial hemorrhages or focal brain ischemia. Accordingly, consideration of focal lesions in the whole-cerebrum densitometric analysis may be restricted, and this study thus focused on the interpretation of TBI pathologies involved in global parenchymal changes.

### Quantitative Assessment of Secondary Ischemic-Edematous Insults in the Acute Phase of TBI

Intracranial densitometry offers a wide variety of quantitative parameters, i.e., morphological parameters (μ, skewness, and kurtosis) and proportional parameters (dWx, dGx, and cEs). The morphological parameters are derived from a whole-cerebrum density distribution (Figure 3). Based on the dichotomized outcome favorability, the averaged density distribution of patients with an unfavorable outcome was determined to be significantly right-skewed in distribution with a leftward shift of the center compared to that of patients with a favorable outcome. The distortion of the intracranial densitometry in the unfavorable outcome group became further apparent in the follow-up CT. These morphological characteristics are reflected as an increase in skewness and a decrease in μ, respectively, and suggest that the overall density of the parenchyma was reduced due to a subtle global ischemic change after TBI (37). The unfavorable outcome group also showed a higher kurtosis value in their density distributions than the favorable outcome group. Kurtosis, which has often been wrongfully interpreted as a measure of the “peakedness” of distribution graphs, is actually a measure of outliers (40). Localized hyper- or hypodense lesions result in significant increases in a specific range of HU values, i.e., they are outliers that contribute to increased kurtosis of the density distribution (Table 2). Although the statistical power was limited due to the small number of subjects in the follow-up CT subgroup, this group exhibited the same trend as the outcome group in terms of the morphological parameters from the initial CT. These morphological parameters were the main contributors to the outcome prediction of the proposed model (Figure 5).

Unlike morphological parameters, which mainly reflect whole-cerebrum density distribution, proportional parameters reflect the density distribution of specific, major intracranial entities, and can be interpreted in relation to the pathophysiology of cerebral edema. Cerebral edema is divided into vasogenic and cytotoxic types. In general, cytotoxic edema affects both WM and GM (41), whereas vasogenic edema primarily affects WM and easily spreads to other locations via WM tracts (42). In the acute phase of TBI, vasogenic, and cytotoxic edema often coexist (43). Thus, reduced brain tissue density mediated by cerebral edema can be simultaneously reflected by lower WM and GM density values on CT. The increased proportion of hypodense pixels in CT images is reflected as an increase in the cEs (18), whereas changes in WM and GM densities are reflected as changes in dWx and dGx (37). Intriguingly, the conventional statistical analysis indicated that only cEs was significant in differentiating favorable and unfavorable outcome groups based on the initial CT (Table 2), whereas the machine learning model indicated that dGx had the highest importance among the proportional parameters, followed by cEs and dWx (Figure 5). Nonetheless, inferential statistics are influenced by effect size or sample size (44), and statistical significance does not guarantee high feature importance in the prognostic model. Indeed, dGx, the proportion of normal-density GM, which did not differ significantly between the two outcome groups, was considered a significant predictor of the outcome by the utilized machine learning model. Nevertheless, in follow-up CT within 48 h, dGx showed a significant difference between the outcome favorability. Despite the shortage of statistical power, this would suggest that brain density alternation even affected the relative proportion of GM with normal density.

In addition to the densitometric analysis, quantitative CT classification systems (i.e., Marshall and Rotterdam score) have been evaluated for use in assessing the severity of TBI. In pediatric TBI, the Rotterdam score has better discriminatory power than that of Marshall (45). However, neither the Marshall nor the Rotterdam scores classified outcomes well in our cohort (Tables 2, 4), which may be attributed to the cohort characteristics of this pilot study. Originally, the Rotterdam score was developed for only moderate or severe TBI, excluding mild head injury (10). Liesemer et al. reported that although the Rotterdam score was initially developed for use in the adult population, the prognostic function worked well in the pediatric TBI population (46); however, ~80% of the subjects had moderate or severe TBI (GCS, 3–8). A study in which the Marshall score showed adequate prognostic ability in pediatric TBI was also composed of moderate or severe TBI in 60% of the cohort (45). Consequently, the conventional CT classification systems could not work properly in this study, where ~70% of mild TBI patients are composed.

### Responsiveness of Intracranial Densitometry to Alternative Outcome Measures

Mortality, LOS, and the need for surgical procedures are primary outcome measures for pediatric TBI in clinical practice (47). In addition to outcome favorability, we investigated how intracranial densitometry responds to these outcome measures. LOS was closely associated with mortality in TBI patients (48); nevertheless, mortality is the worst consequence of TBI. Therefore, undoubtedly, the most sensitive response of intracranial densitometry was mortality. The averaged densitometry between the survival and deceased groups showed the most distinguishable difference among the alternative outcome measures. This finding suggests that deceased patients enter an irreversible state in which the brain densitometry was significantly different from the normal state. On the other hand, the morphology of intracranial densitometry was relatively similar between groups based on the LOS, a less severe outcome. However, the dichotomized LOS revealed a significant difference in the GM-related HU range. In contrast to mortality and LOS, no statistically significant difference in intracranial densitometry was observed between the groups classified by surgery necessity. This non-significance implies that, as mentioned above, the focal lesions that require surgical intervention may have a limited effect on the deformation of whole-cerebrum densitometry.

### Machine-Learning-Based Prognostic TBI Model Augmented by Densitometric Information

This study proposed a novel prognostic model based on GDBTs augmented with CRASH-CT and intracranial densitometric information. Based on various outcome measures, the proposed models (i.e., D/CRASH-CT_CatBoost_) achieved enhanced prognostic capacity compared with the conventional model; this consistent improvement can be contributed by a combination of (1) densitometric information and (2) machine learning models. Intracranial densitometry is a quantitative input variable that responds to global pathological changes in the intracranial region (18, 25). The proposed model included additional densitometric information, enhancing prognostic values, especially for GOS-related outcome measures (Figures 5, 6A). On the other hand, in terms of LOS and surgery necessity, CRASH-CT input variables (e.g., age, GCS, pupil reaction, extracranial injury, CT findings) played a more substantial contribution than the densitometric information (Figures 6B,C). In this case, it can be assumed that machine learning itself contributed to increasing the prognostic value rather than adding the densitometric variables. Logistic regression does not consider the correlation between the input variables and has multicollinearity problems (49), whereas CatBoost can lead to better performance by reducing information loss by creating a combination considering the correlation between the input variables (50).

The significant factors contributing to the decreased prognostic capacity of the conventional CRASH-CT model are 2-fold, namely, interrater variability, and validation method. The CRASH-CT model is a widely used prognostic TBI model (13), and its variables are based on the injury status and initial CT findings. The radiological findings used as the input of the CRASH-CT model are based on the Marshall CT classification, which has been reported to have ~12.7% interrater variability (51). Interrater variability changes the results of a prognostic model and thus lowers its reliability. In addition, CRASH-CT yielded low accuracy for pediatric TBI patients in this study, unlike reports in the literature (25, 52). It can be assumed that it is not properly fitted to predict the outcome at discharge since the original CRASH-CT model predicts outcome favorability after 14 days (13). In addition, the LOOCV method, a stricter validation method than others reported in the literature (25, 52), also contributed to the low prognostic capacity of the CRASH-CT model. Previous studies (13, 25, 52) in which the training and testing of logistic regression models were performed with the same cohort without external validation have the potential to overestimate the prognostic capacity, and it is complicated to resolve the overfitting problem oriented to the cohort. In this study, individual and iterative validations were performed 58 times by separating training and testing subjects through LOOCV. Despite the more rigorously evaluated results, improvements in prognostic value were observed by adding densitometric information to the CRASH-CT input. A significantly enhanced prognostic value could be achieved compared with CRASH-CT when only initial brain CT data were used, suggesting that machine learning-based automated densitometry is a useful prognostic tool that can minimize unnecessary radiation exposure in children with TBI.

In several recent studies, prognostic models using machine learning have been proposed to predict the outcome favorability of patients with pediatric TBI more accurately (53–55). Kayhanian et al. proposed a support vector machine (SVM)-based prognostic model using admission laboratory variables (53). Hale et al. developed an artificial neural network (ANN) model using laboratory values, GCS scores, and initial CT findings (54). Tunthanatip et al. compared the prognostic value of various machine learning models using comorbidity and radiological finding information and concluded that the SVM-based model showed the highest performance (55). These studies consistently reported that machine learning outperformed conventional logistic regression (53–55), and the same results were obtained in the present study. However, unlike the GBDT used in this study, the ANN and SVM models used in previous studies are “black box” models that are difficult to interpret (56). Interpretability of the model is an important issue when using machine learning in the medical domain (21). A black box-based prognostic model that lacks an explanation of how much an input variable contributed to the prediction may have limited application in the clinical environment (57). It is complicated to convince a clinician of the results of a model when they are presented without any explanation. On the other hand, the proposed GBDT-based model showed reliable prognostic capacity and could explain how the input variables contribute to the prediction. Consequently, unlike the previous ML-based prognostic models for pediatric TBI, which entail a trade-off between interpretability and prognostic capacity (53–55), the proposed model accomplished both.

### Limitations and Suggestions

Several limitations should be considered. First, this study was a single-center, retrospective pilot study with a small cohort size. The small cohort size hampered the appropriate distribution of TBI severity; mild TBI patients were the most dominant in this study. Thus, the proposed model should be validated by a large-scale dataset, and a prospective, multicenter study is required for generalization of the model proposed in this study. Second, this study used only the in-hospital outcome measures because there was no long-term outcome information in our institutional database. The model's prediction of long-term outcomes should also be validated. Third, except for intracranial densitometry, elaborative modalities for assessing cerebral edema in TBI patients were not used in this study. The extent of cerebral edema would have been better assessed and cross-validated using MRI (e.g., diffusion-weighted imaging and apparent diffusion coefficient mapping). In future validation studies, the benefits of the proposed method will be investigated to address the following issues: (1) changes in physician decision-making as the result of utilizing densitometric analysis; (2) estimation of other intracranial pathologies (e.g., intracranial hypertension); and (3) prognostic capacity of the proposed models for predicting long-term outcomes.

## Conclusion

This study revealed that intracranial densitometric information derived from initially acquired brain CT scans performed at admission was highly associated with worse outcomes in pediatric TBI patients. Contrary to the conventional TBI prognostic model (i.e., CRASH-CT model), which mainly uses arbitrary measures (i.e., Marshall classification) that suffer from inter- and intrarater variability, fully automated densitometric analysis of the whole cerebrum was supplemented in the construction of the prognostic model. Accordingly, the prognostic value of brain CT was significantly enhanced by augmenting densitometric information with a cutting-edge GBDT-based machine learning model. In conclusion, intracranial densitometry information could improve the reliability of brain CT-based clinical decision-making during the acute phase of TBI and may serve as the basis for enhancing the TBI prognostic model.

## Data Availability Statement

The datasets presented in this article are not readily available because sharing data outside is not available according to the policy of our institution. Requests to access the datasets should be directed to http://hrpp.snuh.org/.

## Ethics Statement

The studies involving human participants were reviewed and approved by Ethics Committee of Seoul National University Hospital (IRB H-1706-144-862). Written informed consent from the participants' legal guardian/next of kin was not required to participate in this study in accordance with the national legislation and the institutional requirements.

## Author Contributions

Y-TK drafted and revised the manuscript, carried out clinical data analysis and interpretation, and contributed to the design of the study. HK critically revised the manuscript and contributed to the design of the study. C-HL performed prognostic modeling via machine learning. C-HL, BY, JK, YC, W-SC, and B-MO critically revised the manuscript. B-MO performed data collection. D-JK conceptualized and designed the study, critically revised the manuscript, and oversaw the creation of the final manuscript. All authors approved the final manuscript as submitted and agree to be accountable for all aspects of the work.

## Funding

This work was supported by the National Research Foundation of Korea (NRF) grant funded by the Korea government (Ministry of Science and ICT, MSIT) (Grant number 2019R1A2C1003399 and Grant number 2020R1C1C1006773) and a grant of the Korea Health Technology R&D Project through the Korea Health Industry Development Institute (KHIDI) funded by the Ministry of Health & Welfare, Republic of Korea (grant number: HI17C1561).

## Conflict of Interest

The authors declare that the research was conducted in the absence of any commercial or financial relationships that could be construed as a potential conflict of interest.

## Publisher's Note

All claims expressed in this article are solely those of the authors and do not necessarily represent those of their affiliated organizations, or those of the publisher, the editors and the reviewers. Any product that may be evaluated in this article, or claim that may be made by its manufacturer, is not guaranteed or endorsed by the publisher.

## Abbreviations

ANN: artificial neural network
cEs: cerebral edema score
CRASH: corticosteroid randomization after significant head injury
CT: computed tomography
dGx: proportions of normal-density gray matter
dWx: proportions of normal-density white matter
GBDT: gradient-boosted decision tree
GM: gray matter
HU: Hounsfield unit
IMPACT: international mission for prognosis and analysis of clinical trials
LOOCV: leave-one-out cross-validation
MRI: magnetic resonance imaging
PCA: principal component analysis
SHAP: Shapley additional explanations
SVM: support vector machine
TBI: traumatic brain injury
WM: white matter.
